# Utility of intranasal Ketamine and Midazolam to perform gastric aspirates in children: a double-blind, placebo controlled, randomized study

**DOI:** 10.1186/1471-2431-14-67

**Published:** 2014-03-05

**Authors:** Danilo Buonsenso, Giovanni Barone, Piero Valentini, Filomena Pierri, Riccardo Riccardi, Antonio Chiaretti

**Affiliations:** 1 Department of Pediatric Sciences, Catholic University of Rome, Largo A. Gemelli, 1, 00168 Rome, Italy

**Keywords:** Intranasal sedation, Midazolam, Ketamine, Tuberculosis, Gastric washings

## Abstract

**Background:**

We performed a prospective, randomized, placebo-controlled study aimed to evaluate the efficacy and safety of a sedation protocol based on intranasal Ketamine and Midazolam (INKM) administered by a mucosal atomizer device in uncooperative children undergoing gastric aspirates for suspected tuberculosis. *Primary outcome:* evaluation of Modified Objective Pain Score (MOPS) reduction in children undergoing INKM compared to the placebo group. *Secondary outcomes*: evaluation of safety of INKM protocol, start time sedation effect, duration of sedation and evaluation of parents and doctors’ satisfaction about the procedure.

**Methods:**

In the sedation group, 19 children, mean age 41.5 months, received intranasal Midazolam (0.5 mg/kg) and Ketamine (2 mg/kg). In the placebo group, 17 children received normal saline solution twice in each nostril. The child’s degree of sedation was scored using the MOPS. A questionnaire was designed to evaluate the parents’ and doctors’ opinions on the procedures of both groups.

**Results:**

Fifty-seven gastric washings were performed in the sedation-group, while in the placebo-group we performed 51 gastric aspirates. The degree of sedation achieved by INMK enabled all procedures to be completed without additional drugs. The mean duration of sedation was 71.5 min. Mean MOPS was 3.5 (range 1-8) in the sedation-group, 7.2 (range 4-9) in the placebo-group (p <0.0001). The questionnaire revealed high levels of satisfaction by both doctors and parents in the sedation-group compared to the placebo-group. The only side effect registered was post-sedation agitation in 6 procedures in the sedation group (10.5%).

**Conclusions:**

Our experience suggests that atomized INKM makes gastric aspirates more acceptable and easy to perform in children.

**Trial registration:**

Unique trial Number: UMIN000010623; Receipt Number: R000012422.

## Background

Tuberculosis (TB) is among the top 10 causes of childhood death worldwide. Because children with pulmonary TB are unable to produce sputum, gastric aspirate is the procedure of choice for microbiologic confirmation of disease. Attention to the technique of gastric aspirate collection has been demonstrated to improve the yield of this diagnostic tool [1], but this procedure requires children to be cooperative and a noninvasive route for sedative drug administration, such as intranasal, may be useful in obtaining a short-time sedation. This is particularly important if we consider that nasogastric tube insertion (NGTI) is one of the most painful and uncomfortable procedures performed in children [2-5]. Several methods have been proposed to reduce the discomfort of NGTI, such as using lubricant gel, topical Lidocaine sprays, Lidocaine gel as well as other non-medical strategies [6], but none showed a strong benefit, particularly in children. In fact, despite all these methods, NGTI following the administration of water-soluble lubricating gel is still one of the most used methods in many medical centers [7,8]. Intranasal Midazolam (INM) has been found to be effective in doses ranging from 0.2 to 0.5 mg/kg when used for conscious sedation [9]. The intranasal route is preferable since it obviates the need for intravenous access, is easily accessible and allow a more rapid rate of absorption compared to the oral route [10,11]. Although most studies investigating INM administered the drug by drop instillation, new methods such as spray devices are being explored. Atomization devices were assessed in several studies and showed good tolerance, safety and efficacy [12]. A Mucosal Atomizer Device (MAD) delivers medications via a fine spray over a broad surface area in the nasal cavity. It also reduces sneezing and coughing compared to other devices [9]. Ketamine has local anesthetic properties and interacts with the N-methyl-D-aspartate receptors both in the vascular endothelium and in the central nervous system, thus attenuating the afferent and central pain pathways [13,14]. Its activity has some advantages, such as preservation of respiratory reflexes and an intrinsic positive inotropic effect [15]. It is an excellent analgesic, sedative and amnestic agent. Midazolam-Ketamine combination has been used for different pediatric procedures inside and outside the operating room, for its anxiolytic and analgesic effects, in order to obtain more analgesia, less hypotension, the use of a lower doses of drugs, and, consequently, a lower risk of respiratory depression [16].

In this study we prospectively evaluated the efficacy and safety of a sedation protocol based on intranasal combination of Ketamine and Midazolam (INKM) administered by MAD, in children undergoing gastric aspirates for suspected pulmonary TB. The *primary outcome* was to evaluate MOPS reduction in children undergoing INKM compared to the placebo group. *Secondary outcomes* were: evaluation of safety of INKM protocol, start time sedation effect, duration of sedation and evaluation of parents and doctors’ satisfaction about the procedure.

## Methods

We performed a prospective, placebo-controlled, randomized, pilot study aimed to evaluate the efficacy and safety of INKM in uncooperative children undergoing gastric aspirates for suspected TB, in children admitted to the pediatric infectious diseases unit of our Institution (Unique trial Number: UMIN000010623; Receipt Number: R000012422) [17].

**Figure 1:**
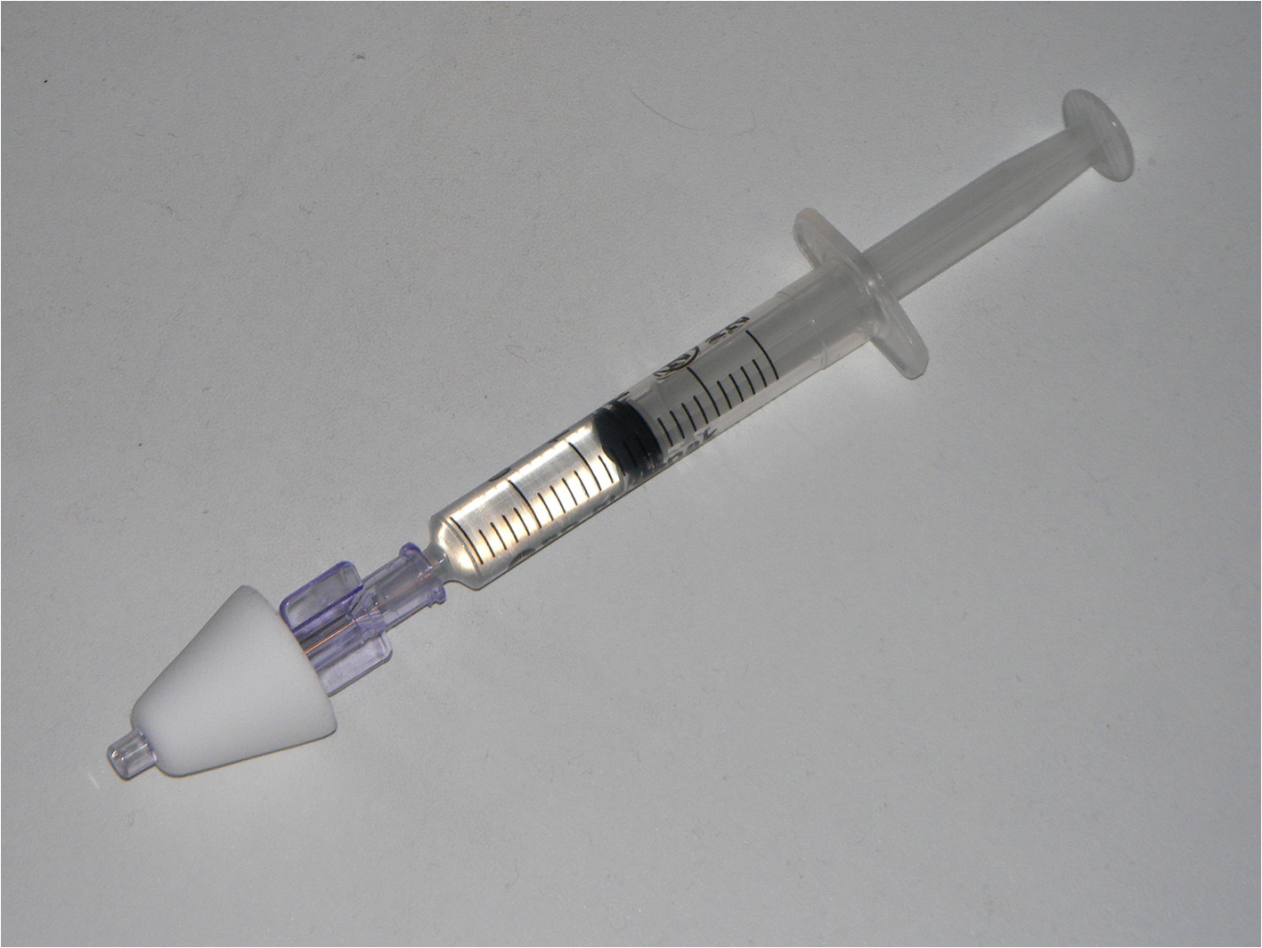
The Mucosal Atomiser Device (MAD) used to deliver medications via a fine spray in the nasal cavity.

**Figure 2:**
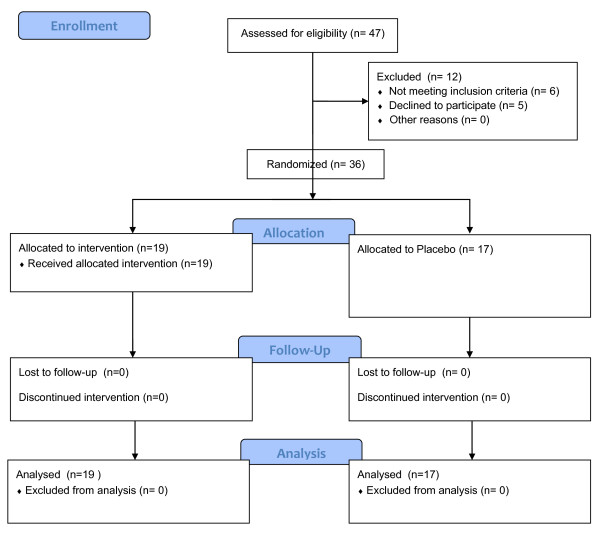
Patients allocation (CONSORT).

Pre-sedation behavior was assessed on a 4-point scale (1 = calm, cooperative; 2 = anxious but reassurable; 3 = anxious and not reassurable; 4 = crying or resisting) by an anesthesiologist who was blinded to the group of the child. Children were included if they were <14 years old and had a pre-sedation behavior ≥ 3. Children were excluded if they had an ASA classification of III or higher, a known allergy to benzodiazepines, an upper respiratory tract infection with nasal discharge, a known liver disease or respiratory distress, known allergy to ketamine, and age > 14 years (168 months). An independent investigator, blind in both sedation and placebo group and not involved in the observation or providing anesthesia to the child, prepared the syringes for the two groups. In the sedation-group, children received 2 mg/kg of Ketamine hydrochloride administered by a physician in both nostrils followed by 0.5 mg/kg (maximum dose 10 mg = 2 mL) of Midazolam using a 2.5 mL syringe connected to a MAD (Wolfe Tory Medical, Salt Lake City, Utah, USA) (Figure 1). In the placebo-group, children received normal saline solution (the same volume the child would have received if in the sedation-group) in each nostril (twice, in order to pretend the two different drugs of the sedation-group) (Figure 2). Drugs were administered by a blinded doctor not involved in syringe preparation. Patients were randomly assigned to one of two groups according to a computer-generated randomization number enclosed in sequentially numbered envelopes which were opened just before the procedure and according with CONSORT statement [17]. Gastric washings were performed on three consecutive days early in the morning and after an overnight fasting. An unbiased doctor (not involved in pre-sedation behaviour assessment, nor in syringes preparation) inserted a nasogastric tube into the child’s stomach after intranasal drug/placebo administration (without any additional drugs nor topical anaesthetics on nasogastic tube) and then aspirated its contents; in case of unsuccessful or poor aspiration, the volume of gastric aspirates was augmented as needed by injecting in the stomach 5 mL of saline solution (sterile water) and aspirating back [18]. The procedure began in every case within 60 minutes from intranasal administration (either drug or placebo); this fix time-frame was pre-established because from our personal experience (data not shown) sedation effect always began within 60 minutes from drug administration. Bloodpressure was measured at baseline and at 5 min intervals. Pulse oximetry was continuously monitored and the lowest oxygen saturation (O_2_ sat) was recorded. ‘Start time’ (sedation effect onset), ‘end time’ (end of the sedation effect) and duration of sedation (difference between the end and start times) were registered. The child’s degree of sedation and reactivity during the procedures were scored by an unbiased doctor, not involved in pre-sedation behaviour assessment, nor in syringes preparation, and nor in NGTI. This doctor used the Modified Objective Pain Score (MOPS), modified for children under 2 years of age, according to author’s indications, which is also appropriate for children aged 2-11 years. MOPS has already been tested for both post-operative and non post-operative pain in children [19,20].

The score ranges from 0 to 10 (the higher the score, the greater the pain experienced for the child). Drug efficacy, safety, tolerability and procedure times were recorded by an unbiased blinded doctor who did not participate in the sedation and procedures. Risks, possible discomforts and benefits were explained to the parents and they were required to sign an informed consent form prior to the procedure. At the end of sedation, the unbiased blinded doctor submitted a questionnaire to the medical staff who did not participate in the sedation but performed the painful procedure, and to the patient’s parents. Physicians and parents of both groups indicated on a visual analogue scale (‘0’ for worst, ‘10’ for best) the usefulness of the drug, changes in the child’s and parents’ outlooks and whether they would recommend the sedation by MAD to other physicians and parents. The same unbiased blinded doctor evaluated all procedures in order to minimize inter observed variability. Informed consent was obtained from every child’ parent. Ethics approval was obtained from the Ethics Committee of our Hospital.

### Statistical analysis

Univariate linear regression testing was used to measure the correlation of MOPS, start time, end time and length of sedation with the number of procedures, children’s weight and age. MOPS in the sedation-group was compared with MOPS in the placebo-group by using unpaired t-test. A *P* value <0.05 was considered significant. Statistical analysis was performed using SPSS v 17. MOPS was the primary outcome of this study, which allowed the sample size to be calculated on that basis. A retrospective review of our database concerning gastric aspirates for TB in the previous 2 years yielded a MOPS (mean ± SD) of 7 ± 2. Calculations derived a sample size of 17 children in each group to yield 80% power (with alfa .05) in detecting a reduction of 2 point in MOPS. Secondary outcomes were: evaluation of safety of INKM protocol, start time sedation effect, duration of sedation and evaluation of parents and doctors’ satisfaction about the procedure.

## Results

From January 1, 2009 to July 31, 2012 a total of 27 patients were recruited to the study. The sedation-group consisted of 19 (8 males, 11 females) children (mean age 41.5 months, IC95% 24.2 to 58.9; median age 31 months, IC 95% 18.8 to 49.3, SD 36.0, min 13, max 168) (Table 1); fifty-seven gastric washings were performed in these children (one child underwent the procedure twice). Fifteen children were diagnosed with pulmonary TB, one with latent TB infection and three with non-TB pneumonia. The placebo-group consisted of 17 (7 males, 10 females) children (mean age 40.6 months, IC 95% 21.3 to 59.9; median age 33 months, IC 95% 19.2 to 48.7, SD 34.8, min 14, max 152), all with a final diagnosis of active TB; fifty-one gastric aspirates were performed in this group. The two groups did not differ significantly with respect to age, weight and ethnicity.

**Table 1:** Age, start times, end times and duration of the sedation effect in children undergoing procedural sedation by intranasal Midazolam and Ketamine and MOPS achieved in both the sedation and placebo groups

**Parameter**	**Mean**	**IC 95%**	**Median**	**IC 95%**	**Standard deviation**	**Min**	**Max**
**Age (mths) sedation**	41.5	24.2 to 58.9	31.0	18.8 to 49.3	36.0	13	168
**Age (mnths) placebo**	40.6	21.3 to 59.9	33.0	19.2 to 48.7	34.8	14	152
**Start time sedation**	22.9	19.2 to 26.6	24	15.4 to 26.6	13.7	6	60
**Effect (min) sedation**							
**Start time sedation**	Na^#^	na	na	na	na	na	
**Effect (min) placebo**							
**End time sedation effect (min) sedation**	77.9	70.1 to 85.7	80	75.0 to 84.0	29.2	30	180
**End time sedation effect (min) placebo**	Na	na	na	na	na	na	Na
**Duration of sedation effect (min) sedation**	71.5	63.8 to 79.3	72.0	65.6 to 76.6	28.9	25	176
**Duration of sedation effect (min) placebo**	Na	na	na	na	na	na	Na
MOPS sedation* (IQR)	3.5	3.1 to 3.9	4.0	3.0 to 4.0	1.6	1	8
							**(2.5-4)**
MOPS placebo* (IQR)	7.2	6.9 to 7.6	7	7.0 to 8.0	1.4	4	9
							**(6-8)**

*p <0.0001.

^#^na: not applicable due to no effects of placebo.

### Sedation-group

The mean time between administering INKM and the beginning of the effect was 22.9 min. The mean time between administering INKM and the end of the sedation effect was 77.9 min. The mean duration of the sedation after INKM effect was 71.5 min. Mean time needed to complete the procedure was 7 minutes (range, 4-10 minutes). Mean MOPS was 3.5, **interquartile range 2.5 - 4** (Table 1). The degree of sedation achieved enabled to perform all physician inserting NG tube and aspirating gastric content, without the need of a second doctor/nurse for physical restrain. In two cases (3.7% of all 57 procedures), the level of sedation obtained was low (7 and 8, respectively) and a nurse was needed for physical restrain. The only side effect registered was transitory postsedation agitation in 6 out of 57 procedures (10.5%). O2 sat levels ranged between 96% and 100% during all time of procedure.

### Placebo-group

After a mean time of 60.3 minutes from the administration of normal saline solution in each nostril, no change in children behavior was noted in all cases and the blinded doctor began the procedure. Mean MOPS score was 7.2 (SD 1.4, range 4-9, interquartile range 6-8) (Table 1). Mean time needed to complete the procedure was 7.5 minutes (range, 5-9.8). None of the fifty-one procedures could be performed by only one doctor, 15 (29.5%) procedures had to be performed by two operators (one doctor needed to perform the gastric aspirate, one nurse for physical restrain), 36 (70.5%) procedures had to be performed by three operators (one doctor to perform the gastric aspirate, two nurses for physical restrain); during these 36 procedures, children needed to be wrapped up in bed sheets to ensure the child to be blocked and the correct execution of the procedure.

### Patients’ parents and doctors’ appreciation

Patients’ parents of both groups blindly reported their appreciation of the ease and utility of intranasal drug administration to perform gastric aspirates. Both parents and medical staff reported a major level of satisfaction regarding the children’s behavior during the procedures in the sedation-group respect to the placebo group (Table 2).

**Table 2:** Parents’ and medical doctors’ responses to the questionnaire on the administration of intranasal Ketamine and Midazolam (table A) and placebo (table B) via a mucosal atomiser device

**A**						
**Question**	**Parents (n)**	**Score (median)**	**Range**	Doctors* (n)	**Score (median)**	**Range**
**The use of INMK by MAD:**						
Helped	19	10	10-10	5/3	10	10-10
Level of child’s outlook	19	8.9	7-10	5/3	8	7-9
Level of parents’ outlook	19	9.1	8-10	5/3	9.5	9-10
Level of doctors’ outlook	-	-		5/3	8.5	8-10
Level of child’s tolerance of procedures	19	8.7	7-10	5/3	8.2	7-9
Would recommend to other parents	19	9.3	9-10	-	-	
Would recommend to other doctors	-	-		5/3	9.4	9-10
Would like to see MAD used routinely	19	9.8	9-10	5/3	10	10
Made the procedure more acceptable	-	-	-	5/3	10	10
**B**						
**Question**	**Parents (n)**	**Score (median)**	**Range**	Doctors* (n)	**Score (median)**	**Range**
**The use of intranasal sedation (placebo) by MAD:**						
Helped	19	5	3-7	5/3	3	2-4
Level of child’s outlook	19	5.8	5-7	5/3	3	2-4
Level of parents’ outlook	19	4.9	3-7	5/3	5	3-7
Level of doctors’ outlook	-	-		5/3	4	3-5
Level of child’s tolerance of procedures	19	8.5	7-10	5/3	8	7-9
Would recommend to other parents	19	4	3-6	-	-	
Would recommend to other doctors	-	-		5/3	3	1-5
Would like to see MAD used routinely	19	4	3-6	5/3	3	1-5
Made the procedure more acceptable	-	-	-	5/3	3	1-5

*unbiased medical doctors involved in the painful or diagnostic procedures (text for details).

INKM, intranasal midazolam and ketamine.

MAD, mucosal atomiser device.

### Statistical analysis

The mean degree of sedation achieved in the sedation-group was statistically higher than that of the placebo-group (p <0.0001). In the sedation-group, we did not find statistically significant correlation between MOPS, start time, end time and duration of sedation with age, weight and number of procedures. The correlation between ethnicity and degree of sedation achieved has not been performed due to the low number of patients evaluated.

### Cost analysis

The preparation of Midazolam (Ipnovel 5 mg/mL, Roche, Milano, Italy) we used (1 mL vial, concentration: 5 mg/mL) has a price of 4.70 euro (6.41 $), which means that 0.5 mg has a theoretical price of 0.47 euro (0.641 $); therefore for every child’s kilogram a purchase of 0.47 euro (0.641 $) would be required. The preparation of Ketamine (Ketamina Molteni 50 mg/mL, Firenze, Italy) we used (2 mL vial, concentration: 50 mg/mL) has a price of 4.916 (6.706 $) euro per vial, which means that 2 mg has a theoretical price of 0.196 euro (0.267 $), therefore for every child’s kilogram a purchase of 0.196 euro (0.267 $) would be required. The MAD we used has a prize for the single device of 3.96 euro (5.36 $). The same device can be used for all the three procedures every single child underwent; after its use, the device need to be thrown away. For example, to sedate a 10 kg child with our protocol, a total purchase of 10.59 euro (14.44 $) (6.66 euro (9.086 $) for drugs plus 3.96 euro (5.36 $) for MAD) would be needed.

## Discussion

This study shows that INKM administered via MAD seems to be effective and safe when gastric washings in anxious and uncooperative children need to be performed to diagnose TB. All patients achieved an adequate level of sedation throughout each procedure, with a statistically significant difference compared to the placebo-group in the degree of MOPS reduction. Both the medical staff and the patient’s parents reported a significant degree of satisfaction with the INKM protocol used to perform gastric aspirates respect to the placebo group. There were no serious side effects, such as oxygen desaturation, bradycardia, hypotension or apnoea. The only side effect registered was post-sedation agitation. As described in literature, the use of Ketamine is known to result in an agitated, confused, and combative child. There has been no documented effective strategy in the literature to manage this problem but these adverse reactions are usually transient and completely recover spontaneously without any adverse consequences [21]. On the other hand, several studies revealed that intranasal Midazolam administered by MAD or by drops resulted in nasal burning and a bitter taste in up to 66% of patients, making the experience unpleasant and the procedure more difficult to complete [22,23]. The administration of intranasal Lidocaine prior to the use of intranasal Midazolam has been reported to be beneficial in reducing the burning sensation from the intranasal Midazolam [11]. In our experience we decided to not use intranasal Lidocaine before the Midazolam administration due to the local anesthetic properties of the Ketamine able to avoid any nasal discomfort related to intranasal Midazolam. Recent studies also reported the use of much higher doses of intranasal Ketamine (up to 9 mg/kg) to perform different painful procedures in pediatric age [24,25]. In our experience we decided to utilize a relatively low dose of Ketamine to evaluate both its efficacy and also to provide a safety dose of the drug able to avoid the more frequent side effects related to its use, such as nausea, vomiting, respiratory depression, and laryngospasm. Recently, it has been reported that high doses of Ketamine in different procedures involving major airways, such as gastroscopy and bronchoscopy, are responsible of the appearance of the laryngospasm in about 10% of cases needed O_2_ ventilation by face-mask and continuous positive airway pressure to be resolved [11,26,27]. Our results showed that, despite the low doses of intranasal Ketamine, we were able to complete all the procedures without major side effects, such as laryngospam, in treated children making this procedure very easy. In fact, the only side effect observed in the sedation group was the post-sedation agitation in 14.3% of cases. Obviously, a slightly higher dose of Ketamine could have induced a higher sedation allowing to complete the procedure by a single physician in every cases, particularly in younger children, since other authors found that younger children required higher dosage in milligrams per kilogram of Ketamine for adequate sedation [28]. This may be explained by the faster metabolism and renal clearance leading to a shorter half-life of Ketamine in children compared to adults [29,30]. Nevertheless, Hosseini Jahromi SA et al. recently found that by increasing the dose of intranasal Ketamine (0.5 mg/kg vs 3 mg/kg), the children developed less sedation than those who received a lower dose of Ketamine [25], suggesting that a dosage as low as 0.5 mg/kg intranasal Ketamine might be suitable for achieving an adequate therapeutic action (sedation and anxiolysis) combined with lesser side effects because there is a dose–response correlation between Ketamine and its side effects [8,14,28]. Similarly, Yeaman F et al. recently demonstrated that sub-dissociative doses of intranasal Ketamine provided adequate analgesia by 30 min for most paediatric patients aged 3-13 years admitted to the emergency department with moderate to severe pain from isolated limb injury [31]. The PICHFORK (Pain In CHildren Fentanyl OR Ketamine) trial [32] aimed to compare the efficacy of intranasal Ketamine and Fentanyl in the relief of moderate to severe pain in children with limb injuries will use low doses of intranasal Ketamine (1 mg/kg). All these findings support our choice of using moderate doses of Ketamine (2 mg/kg) in order to achieve a proper sedative/analgesic effect while reducing the possibility of severe adverse events, making our protocol applicable also in pediatric wards. Moreover, in our study, we did not applied local anesthetics on nasogastric tube, for two reasons. Firstly, in current literature it is not recommended for the specific use of collect samples for the detection of *Mycobacterium tuberculosis* (MTB) in children [18]; secondly, local anesthetics have a well established antimicrobial activity versus bacteria and fungi [33-35]. Although this aspect has not yet been studied versus mycobacteria, the even low risk of potentially reducing the possibility of culture MTB induced us to not apply local anesthetics on nasogastric tubes. Moreover, even though local anesthetics may reduce the pain related to NGTI, it would not change the experience of the child undergoing the procedure. This is particularly important for those cases, such as suspected TB patients, who need to be submitted to frequent NGTIs to perform the diagnosis and for treatment monitoring. The advantage of a procedural sedation over the local anesthetic, in such situations, is also to improve the all experience for both doctors, physicians and parents particularly when a single child need to undergo this procedure more times. Our results confirm all those findings making our INKM-based protocol a simple, safe and affordable way to perform this procedure in uncooperative children. The use of this protocol would allow even a single nurse or physician (after a brief training program) to perform the procedure, ensuring pain relief and patients and parents’ comfort. Our results could be particularly useful in low-income high TB-burden countries, where a big number of gastric washings would need to be performed daily and where there are limited physician and only a few with specific training in procedural sedation [36]. Moreover, our protocol had low drug-related costs and allowed to reduce the number of health care workers necessary to perform the procedure. Nevertheless, these savings should be counterbalanced with costs related to monitoring (both health care workers and equipment). In fact, we found a long median recovery time (79.7 min). The recovery time has wide individual variations and depends on the doses of medications and clearance in the body. However, the relatively long recovery time is not a major problem in ward settings and day hospital regimens.

### Limitations of the study

The first one is the low number of children evaluated. This makes the data difficult to interpret and did not allow us to find any variables (gender, weight, ethnicity, etc) that could be associated with a higher degree of sedation. Moreover, the low number of children did not allow us to strongly affirm the safety of our protocol, although the preliminary results of our study are promising. If our results would be confirmed by a multicentre, prospective, controlled, randomized trial our protocol could be easily introduced in general practice not only for gastric washings in children with suspected TB but also for different kind of procedural sedation in uncooperative children.

Secondly, the subjectivity of pain measurement by both parents and physicians is a clear limitation which is difficult to overcome. Several sedation scoring scales have been described for children but in current literature there are no comparison studies of all scales; nevertheless, the validity of MOPS has been clearly described [19,20]. In this regard, other pain scores have been described in literature. Among those, the FLACC scale have been widely used for post-operative and non post-operative pain assessment, and could have been used also in our study [37]. Nevertheless, we decided to not use FLACC score because the Authors clearly showed that this score can be high during non-painful procedures and during restraint phase of painful procedures, indicating that FLACC scale measures a composite of pain and distress in young children, in this way altering the application of this score in our study [38]. Finally, we analyzed drug-related costs, which are affordable even in low income countries and allow to perform procedures by a lower number of health care workers, but clearly need to be counterbalanced by monitoring and equipment costs, making difficult to establish the cheapest way to perform these procedures. On the other hand, parents appreciation appeared clear. Therefore, the use of the protocol should be evaluated depending on local resources.

## Conclusions

INKM administered by MAD could be a simple and non-invasive approach for the sedation of children undergoing gastric washings and other minor painful procedures or diagnostic investigations. Despite several study limitations, INKM has demonstrated a good level of efficacy and safety in our series. Further studies are needed to clarify the potential of this protocol for the sedation of children in general pediatric departments and emergency rooms, in both high and low-income countries, mainly to better understand the ideal dose of intranasal Ketamine needed to perform effectively different pediatric procedures without serious side effects for the children.

## Competing interests

The authors declare that they have no competing interests.

## Authors’ contributions

AC had primary responsibility for protocol development, patient enrollment, outcome assessment, preliminary data analysis and writing the manuscript. DB and PV participated in the development of the protocol and analytical framework for the study and contributed to the writing of the manuscript. FP performed procedures contributed to the writing of the manuscript. GB performed statistical analyses and contributed to the writing of the manuscript. AC and RR supervised the design and execution of the study, performed the final data analyses and contributed to the writing of the manuscript. All authors read and approved the final manuscript.

## Pre-publication history

The pre-publication history for this paper can be accessed here:

http://www.biomedcentral.com/1471-2431/14/67/prepub

## References

[B1] Zar HJ Hanslo D Apolles P Swingler G Hussey G Induced sputum versus gastric lavage for microbiological confirmation of pulmonary tuberculosis in infants and young children: a prospective study Lancet 2005 365 130 134 10.1016/S0140-6736(05)17702-2 15639294

[B2] Tucker A Lewis J Procedures in practice: passing a nasogastric tube BMJ 1980 281 1128 1129 10.1136/bmj.281.6248.1128 6775756 PMC1714574

[B3] Wrenn K The lowly nasogastric tube: still appropriate after all these years (at times) Am J Emerg Med 1993 11 84 88 10.1016/0735-6757(93)90068-M 8447880

[B4] Morrison R Pain and discomfort associated with common hospital procedures J Pain Symptom Manage 1993 15 91 94 9494307

[B5] Singer AJ Richman PB Kowalska A Thode HC Jr Comparison of patient and practitioner assessments of pain from commonly performed emergency department procedures Ann Emerg Med 1999 33 652 658 10339680

[B6] Juhl FG Conners G Emergency physicians’ practices and attitudes regarding procedural anaesthesia for nasogastric tube insertion Emerg Med J 2005 22 243 245 10.1136/emj.2004.015602 15788820 PMC1726742

[B7] Cullen L Taylor D Taylor S Chu K Nebulized lidocaine decreases the discomfort of nasogastric tube insertion: a randomized, double-blind trial Ann Emerg Med 2004 44 131 137 10.1016/j.annemergmed.2004.03.033 15278085

[B8] Nejati A Golshani K Moradi Lakeh M Khashayar P Moharari RS Ketamine improves nasogastric tube insertion Emerg Med J 2010 27 582 585 10.1136/emj.2009.075275 20360498

[B9] Lloyd CJ Alredy T Lowry JC Intranasal midazolam as an alternative to general anaesthesia in the management of children with oral and maxillofacial trauma Br J Oral Maxillofac Surg 2000 38 593 595 10.1054/bjom.2000.0534 11092772

[B10] Calligaris L Davide Z Alessandra M De Bortoli R Chiaretti A Barbi E Concentrated midazolam for intranasal administration: a pilot study Pediatr Emerg Care 2011 27 245 247 10.1097/PEC.0b013e31820db93b 21378534

[B11] Chiaretti A Barone G Rigante D Ruggiero A Pierri F Barbi E Barone G Riccardi R Intranasal lidocaine and midazolam for procedural sedation in children Arch Dis Child 2011 96 160 163 10.1136/adc.2010.188433 21030365

[B12] Lane RD Schunk JE Atomized intranasal midazolam use for minor procedures in the pediatric emergency department Pediatr Emerg Care 2008 24 300 303 10.1097/PEC.0b013e31816ecb6f 18496113

[B13] Barbi E Marchetti F Gerarduzzi T Neri E Gagliardo A Sarti A Ventura A Pretreatment with intravenous ketamine reduces propofol injection pain Paediatr Anaesth 2003 13 764 768 10.1046/j.1460-9592.2003.01150.x 14617116

[B14] Zahedi H Nikooseresht M Seifrabie M Prevention of propofol injection pain with small-dose ketamine Middle East J Anesthesiol 2009 20 401 404 19950734

[B15] Chiaretti A Ruggiero A Barbi E Pierri F Maurizi P Fantacci C Bersani G Riccardi R Comparison of propofol versus propofol–ketamine combination in pediatric oncologic procedures performed by non-anesthesiologists Pediatr Blood Cancer 2011 57 1163 1167 10.1002/pbc.23170 21584935

[B16] Bahetwar SK Pandey RK Saksena AK Chandra G A comparative evaluation of intranasal midazolam, ketamine and their combination for sedation of young uncooperative pediatric dental patients: a triple blind randomized crossover trial J Clin Pediatr Dent 2011 35 415 420 22046702 10.17796/jcpd.35.4.l43h3354705u2574

[B17] Schulz KF Altman DG Moher D for the CONSORT Group: CONSORT Statement Updated guidelines for reporting parallel group randomised trials Ann Int Med 2010 152 726 732 10.7326/0003-4819-152-11-201006010-00232 20335313

[B18] Oberhelman RA Soto-Castellares G Gilman RH Caviedes L Castillo ME Kolevic L Del Pino T Saito M Salazar-Lindo E Negron E Montenegro S Laguna-Torres VA Moore DA Evans CA Diagnostic approaches for paediatric tuberculosis by use of different specimen types, culture methods, and PCR: a prospective case–control study Lancet Infect Dis 2010 10 612 620 10.1016/S1473-3099(10)70141-9 20656559 PMC2975578

[B19] Wilson GAM Doyle E Validation of three paediatric pain scores for use by parents Anaesthesia 1996 21 1005 1007 8943588 10.1111/j.1365-2044.1996.tb14991.x

[B20] Kenny SE Irvine T Driver CP Nunn AT Losty PD Jones MO Turnock RR Lamont GL Lloyd DA Double blind randomised controlled trial of topical glyceryl trinitrate in anal fissure Arch Dis Child 2001 85 404 407 10.1136/adc.85.5.404 11668104 PMC1718983

[B21] Wathen JE Roback MG Mackenzie T Bothner JP Does midazolam alter the clinical effects of intravenous ketamine sedation in children? A double-blind, randomized, controlled, emergency department trial Ann Emerg Med 2000 36 579 588 10.1067/mem.2000.111131 11097698

[B22] Karl HW Rosenberger JL Larach MG Ruffle JM Transmucosal administration of midazolam for premedication of pediatric patients: comparison of the nasal and sublingual routes Anesthesiology 1993 78 885 891 10.1097/00000542-199305000-00013 8489062

[B23] Hollenhorst J Münte S Friedrich L Heine J Leuwer M Becker H Piepenbrock S Using intranasal midazolam spray to prevent claustrophobia induced by MR imaging AJR Am J Roentgenol 2001 176 865 868 10.2214/ajr.176.4.1760865 11264066

[B24] Tsze DS Steele DW Machan JT Akhlaghi F Linakis JG Intranasal ketamine for procedural sedation in pediatric laceration repair: a preliminary report Pediatr Emerg Care 2012 28 767 770 10.1097/PEC.0b013e3182624935 22858745

[B25] Hosseini Jahromi SA Hosseini Valami SM Adeli N Yazdi Z Comparison of the effects of intranasal midazolam versus different doses of intranasal ketamine on reducing preoperative pediatric anxiety: a prospective randomized clinical trial J Anesth 2012 26 878 8 10.1007/s00540-012-1422-6 22688444

[B26] Melendez E Bachur R Serious adverse events during procedural sedation with ketamine Pediatr Emerg Care 2009 25 325 328 10.1097/PEC.0b013e3181a341e0 19404223

[B27] Burnett AM Watters BJ Barringer KW Griffith KR Frascone RJ Laryngospasm and hypoxia after intramuscular administration of Ketamine to a patient in excited delirium Prehosp Emerg Care 2012 16 412 414 10.3109/10903127.2011.640766 22250698

[B28] Riavis M Laux-End R Carvajal-Busslinger MI Tschappeler H Bianchetti MG Sedation with intravenous benzodiazepine and ketamine for renal biopsies Pediatr Nephrol 1998 12 147 148 10.1007/s004670050426 9543377

[B29] Grant IS Nimmo WS McNicol LR Clements JA Ketamine disposition in children and adults Br J Anaesth 1983 55 1107 1111 10.1093/bja/55.11.1107 6639827

[B30] Green SM Johnson NE Ketamine sedation for pediatric procedures: part 2, review and implications Ann Emerg Med 1990 19 1033 1046 10.1016/S0196-0644(05)82569-7 2203290

[B31] Yeaman F Oakley E Meek R Graudins A Sub-dissociative dose intranasal ketamine for limb injury pain in children in the emergency department: a pilot study Emerg Med Aust 2013 25 161 167 10.1111/1742-6723.12059 23560967

[B32] Graudins A Meek R Egerton-Warburton D Seith R Furness T Chapman R The PICHFORK (Pain InCHildren Fentanyl OR Ketamine) trial comparing the efficacy of intranasal ketamine and fentanyl in the relief of moderate to severe pain in children with limb injuries: study protocol for a randomized controlled trial Trials 2013 14 208 10.1186/1745-6215-14-208 23842536 PMC3716920

[B33] Morrow ME Berry CW Antimicrobial properties of topical anesthetic liquids containing lidocaine or benzocaine Anesth Prog 1988 35 9 13 3278655 PMC2190053

[B34] Rodrigues A Vaz CP Fonseca AF de Oliveira JM Barros H In vitro effect of local anesthetics on candida albicans germ tube formation Infect Dis Obstet Gynecol 1994 1 193 197 10.1155/S1064744994000074 18475344 PMC2364336

[B35] Pelz K Wiedmann-Al-Ahmad M Bogdan C Otten JE Analysis of the antimicrobial activity of local anaesthetics used for dental analgesia J Med Microbiol 2008 57 88 94 10.1099/jmm.0.47339-0 18065672

[B36] Kopf A Patel NB Guide to Pain Management in Low-Resource Settings 2010 Seattle, WA: International Association for the Study of Pain

[B37] Merkel SI Voepel-Lewis T Shayevitz JR Malviya S The FLACC: a behavioral scale for scoring postoperative pain in young children Pediatr Nurs 1997 23 293 297 9220806

[B38] Babl FE Crellin D Cheng J Sullivan TP O’Sullivan R Hutchinson A The use of the faces, legs, activity, cry and consolability scale to assess procedural pain and distress in young children Pediatr Emerg Care 2012 28 1281 1296 10.1097/PEC.0b013e3182767d66 23187981

